# Loss of MMP-27 Predicts Mandibular Bone Invasion in Oral Squamous Cell Carcinoma

**DOI:** 10.3390/cancers14164044

**Published:** 2022-08-22

**Authors:** Jonas Eichberger, Florian Weber, Gerrit Spanier, Michael Gerken, Stephan Schreml, Daniela Schulz, Mathias Fiedler, Nils Ludwig, Richard Josef Bauer, Torsten Eugen Reichert, Tobias Ettl

**Affiliations:** 1 Department of Oral and Maxillofacial Surgery, University Hospital Regensburg, 93053 Regensburg, Germany; 2 Department of Oral and Maxillofacial Surgery and Center for Medical Biotechnology, University Hospital Regensburg, 93053 Regensburg, Germany; 3 Institute of Pathology, University of Regensburg, 93053 Regensburg, Germany; 4 Tumor Center Regensburg, Institute for Quality Assurance and Health Services Research, University of Regensburg, 93053 Regensburg, Germany; 5 Department of Dermatology, University Hospital Regensburg, 93053 Regensburg, Germany

**Keywords:** oral squamous cell carcinoma, OSCC, MMP-27, OPG, RANKL, mandibular bone invasion

## Abstract

**Simple Summary:**

The growth of oral squamous cell carcinoma into the mandible poses significant challenges to head and neck surgery. The resulting need for extensive procedures has a decisive influence on subsequent esthetics and function and therefore also on the patient’s quality of life. The molecular mechanism behind this remains obscure to date. Hence, we investigated the influence of MMP-27, Osteoprotegerin and RANKL, three proteins with importance in bone remodeling. The results showed that tumors exhibited less bone-invasive behavior in the presence of MMP-27. This may be an incentive for further studies to elucidate the molecular mechanisms of mandibular bone invasion in OSCC.

**Abstract:**

Invasion of the mandibular bone is frequent in oral squamous cell carcinoma (OSCC), which often results in extensive ablative and reconstructive procedures for the patient. The purpose of this single-center, retrospective study was to identify and evaluate potential biomarkers and risk factors for bone invasion in OSCC. Initially, *in silico* gene expression analysis was performed for different HNSCC tumor T-stages to find factors associated with invasive (T4a) tumor growth. Afterwards, the protein expression of bone-metabolizing MMP-27, TNFRSF11B (Osteoprotegerin, OPG), and TNFSF11 (RANKL) was investigated via Tissue Microarrays (TMAs) for their impact on mandibular bone invasion. TMAs were assembled from the bone–tumor interface of primary OSCCs of the floor of the mouth and gingiva from 119 patients. Sixty-four carcinomas with patho-histological jaw invasion (pT4a) were compared to 55 carcinomas growing along the mandible without invasion (pT2, pT3). Tissue samples were additionally evaluated for patterns of invasion using the WPOI grading system. Statistical analysis of *in silico* data revealed decreased MMP-27 mRNA expression to be strongly associated with the pT4a-stage in OSCC, indicating invasive tumor growth with infiltration of adjacent anatomical structures. Our own clinico-pathological data on OSCCs presented a significant decrease of MMP-27 in tumors invading the nearby mandible (pT4a), compared to pT2 and pT3 tumors without bone invasion. Loss of MMP27 evolved as the strongest predictor of mandibular bone invasion in binary logistic regression analysis. To our knowledge, this is the first study investigating the role of MMP-27 expression in OSCC and demonstrating the importance of the loss of MMP-27 in mandibular bone invasion.

## 1. Introduction

In 2018, head and neck cancer was listed as the seventh most common form of malignancy worldwide, accounting for more than 880,000 new cases and 453,000 fatalities in that year [1]. Almost half of these cases are attributed to oral cancer, a disease mainly caused by tobacco and alcohol abuse [2]. The treatment is stage-dependent: While surgery is the primary option for stage I or II disease, a combination of surgery with adjuvant radio- (chemo-) therapy is needed in patients with locoregionally advanced disease [3]. Cancerous invasive growth in adjacent anatomical structures is characteristically linked to an increased risk of local, cervical lymphatic, and distant disease recurrence [4]. More than 60% of cases have an advanced stage at the initial presentation, where lymph node metastasis or invasion of adjacent tissue has already occurred [5]. Invasion of the mandibular bone occurs frequently in oral cancer. In cases where clinical and radiographic assessment indicates the possibility of bone involvement, a partial or segmental surgical resection is mandatory, the latter often resulting in cosmetic and functional deficiencies [6]. The prognostic impact of mandibular invasion, however, remains controversial [7,8]. In terms of tumor biology, it remains elusive why some carcinomas of the floor of the mouth grow along the mandibular border, while others invade the mandibular cortex early. There are three types of patterns of bone invasion: the infiltrative form, the erosive form and a combination of both [9,10]. To this day, the molecular drivers of bone infiltration in OSCC remain the subject of research. Physiologically, bone remodeling is regulated by RANK, its ligand RANKL and Osteoprotegerin (OPG), all proteins of the tumor necrosis factor receptor superfamily [11,12]. Activation of the RANK-RANKL axis ultimately leads to activation of the transcription factors NF-κB and NFATc1, resulting in differentiation of precursor cells to osteoclasts [13]. In contrast, OPG is produced by osteoblasts and inhibits osteoclastogenesis by binding to RANKL and subsequently neutralizing its function [14]. Bone remodeling and homeostasis are also influenced by matrix metalloproteinases, a large family of zinc endopeptidases, able to cleave specific extracellular matrices. Currently, 23 MMPs are known to be active in humans [15]. Imbalance of either the RANK/RANKL/OPG-system or dysregulated activity of MMPs can lead to diseases such as osteopetrosis or osteoporosis [16,17]. Shifts in the fine balance of bone remodeling can become sites of attack by aberrant cells, whereby these cells can more easily invade bone due to the disruption of the matrix arrangement and matrix structure. Additionally, RANKL is able to trigger cell migration in epithelial cancer cells [18]. Over the past two decades, the role of RANKL, OPG and various MMPs has been demonstrated in a variety of cancers, including OSCC, where they have been linked to metastasis and bone invasion [19,20,21,22]. MMP-1, MMP-2 and MMP-9 are among the most frequently mutated MMPs in OSCC, where they promote invasiveness as well as metastasis to lymphatic tissue [23,24]. A relatively new member of the MMP family is MMP-27, first discovered in 1998 [25]. Very little is known about MMP-27. Unlike the other proteases, it is not secreted by the cell, nor is it located on the membrane, but remains in the endoplasmic reticulum because of a unique C-terminal domain [26]. The function it performs there is not yet clear. The role of MMP-27 in solid tumors has been discussed but remains obscure [27]. For instance, mutations in the MMP27 gene have been described in melanoma and anaplastic thyroid carcinoma [28,29]. In the present study, *in silico* gene expression analysis of the above-mentioned key players was conducted for OSCCs via the cBioPortal database. Afterwards, an analysis of the expression of MMP-27, RANKL and OPG at the bone-tumor interface was performed by immunohistochemistry (IHC) using tissue microarrays (TMAs) from oral cancer patients who had undergone either partial or segmental resection of the mandible at the University Hospital of Regensburg. The invasive pattern of the tumors was evaluated using the Worst Pattern of Invasion grading system (WPOI).

## 2. Materials and Methods

### 2.1. mRNA Expression Analysis Using the cBioPortal Database

CBioPortal (https://www.cBioPortal.org, accessed on 12 August 2022) is an open web resource that provides a large database of cancer genomics. In this study, mRNA expression profiles of MMP27, TNFRSF11B (Osteoprotegerin or OPG), TNFSF11 (RANKL), MMP1, MMP2 and MMP9 throughout various T-stages (AJCC classification, 7th edition [30]) of head and neck cancer were analyzed and integrated into a T-stage boxplot. For this, Head and Neck Squamous Cell Carcinoma TCGA Firehose Legacy data with 530 tumor samples were selected. Tumor stages T1, T2, T3 and T4a were selected. Primary tumor sites were set as “oral tongue,” “oral cavity,” “floor of mouth,” “buccal mucosa,” “base of tongue,” “alveolar ridge,” “hard palate,” and “lip.” Only primary tumor samples were included, so the number of samples was 305. As next step, the selected database was queried by mRNA expression of either “MMP27,” “TNFRSF11B,” “TNFSF11,” “MMP1, “MMP2,” or “MMP9”; mRNA expression levels of tumor samples were normalized relative to the mRNA contents of diploid tissue. Not all samples were referenced to diploid tissue, so the final number of samples dropped to 298. Additionally, we checked protein expressions of MMP-27, OPG and RANKL for association with T-stage. For this, the data set was queried by protein expression, and only z-scores determined by reverse-phase protein array +/− 1.0 were selected. 300 samples were analyzed for protein expression. Lastly, cBioPortal data were used to explore the correlation of MMP27-, RANKL- and OPG-expression and to investigate a possible impact of those genes on the prognosis of certain patient groups. As the last step, we divided the online cohort into two groups for MMP27, RANKL and OPG, based on the median. We then compared both groups, the bottom half with low mRNA expression and the upper half with high mRNA expression, with respect to each T-stage. Lastly, *in silico* data were analyzed using the Kaplan–Meier method to make assertions about the prognosis of those two groups. 

### 2.2. Patients

Clinical and histopathological data of 151 OSCC patients who underwent surgery and received partial or segmental resection of the mandibular bone in the Department of Oral and Maxillofacial surgery at the University Hospital of Regensburg were collected. The time of diagnosis was between January 2004 and July 2019. The median follow-up time was 7.6 years (95% CI: 5.5–9.7 years), with a maximum follow-up of 17.3 years. Due to missing data or non-retrievable histopathological samples, 32 patients were excluded from this study, resulting in a final number of 119 individuals. The characteristics are summarized in Table 1. All samples were derived from OSCCs growing in close contact with the mandible and originating from the floor of the mouth or the lower alveolus gingiva. Malignancies that developed from adjacent anatomic regions, such as the maxilla or oropharyngeal carcinomas, were excluded. Tumors diagnosed at a very early stage (Tis, T1) were also not included in this study.

### 2.3. Assessment of Tumor Invasion

The pattern of invasion of the tumor front in histological sections was classified using the WPOI grading system (Worst Pattern of Invasion) [31]. Hematoxylin and eosin (H&E) stained slides were analyzed for the extent of invasion into adjacent tissue and the mandible if microscopic bone involvement was observed. Tumors with WPOI grades of 1–3 tend to exhibit non-aggressive behavior and consist of broad, massive thrust fronts, whereas OSCCs with WPOI grades of 4–5 tend to have small satellites that may be separated from the main tumor. In the past, this risk assessment system was highly associated with overall survival and locoregional recurrence in OSCC patients [32].

### 2.4. Immunohistochemical Sample Preparation and Staining Protocol

To perform immunohistochemical staining procedures on the collected samples, a tissue microarray (TMA) was assembled [33]. For each patient, three tissue cylinders with a diameter of 1.5 mm from different areas of the bone–tumor interface were collected, resulting in a total sample size of 357.60 tissue specimens placed in each paraffin block. TMAs were then cut into 3 mm-thick sections and mounted onto Superfrost^®^ Plus Microscope Slides (Thermo Scientific, Waltham, MA, USA). Slides were deparaffinized overnight at 37 °C and rehydrated via serial passage through xylene (three times, 10 min each) and a downward-graded series of alcohol. Antigen retrieval was performed by heating in a Decloaking chamber™ (Biocare Medical, Concord, CA, USA) in TRIS/EDTA buffer for 5 min at 120 °C and 15 bars. Next, the endogenous peroxidase was blocked with Peroxidase Blocking Solution (Dako, Glostrup, Denmark), and antibody incubation was performed by using the following antibodies: anti-MMP-27 polyclonal anti-rabbit IgG antibody (Thermo Scientific, Waltham, MA, USA), diluted to a concentration of 1:100, TNFRSF11B (OPG) polyclonal anti-rabbit IgG antibody (Thermo Scientific, Waltham, MA, USA), diluted to a concentration of 1:100, and CD254 (RANKL) polyclonal anti-rabbit IgG antibody (Thermo Scientific, Waltham, MA, USA), diluted to a concentration of 1:200. All antibodies were incubated for 30 min at room temperature. Staining was performed using the Dako EnVision+™ Detection System, Peroxidase/DAB+, rabbit/mouse (Dako, Glostrup, Denmark). Finally, the slides were counterstained with hematoxylin.

### 2.5. IHC Staining Assessment

The assessment and analysis of staining were performed by an experienced pathologist (F.W.) and a trained investigator (J.E.); in the case of equivocal results, a consensus was reached by joint microscopic evaluation. For MMP-27, both membraneous and cytoplasmic staining were observed, which were analyzed by calculating McCarty’s H-Score [34]. The H-Score system incorporates both the percentage of positive cells and the staining intensity. The relative intensity was defined as non-present (0), weak (1), distinct (2) and strong (3). The cutoff value for high MMP-27 expression was determined as an H-Score over the median of 170.0 (range 5.0–300.0). The cutoff value for low expression was defined to be below the median of 170.0. For TNFRSF11B (OPG), the H-Score was also used for analysis. Cells showed positivity for OPG in the cytoplasm and nucleus, which were analyzed separately. Cytoplasmic stains were considered to have a high expression when the H-Score was more than the median of 40.0 (range 5.0–285.0). Nuclear OPG expression was rated as high when the H-Score was more than 70.0 (range 5.0–180.0). For RANKL, the analysis was carried out according to Quattrini et al. and was slightly modified [35]. The percentage of stained cells was assessed and then divided into four categories (0 = 0%, 1 = 1–10%, 2 = 11–50%, 3 ≥ 50%). Samples with a staining result of 2 or more were considered positive. Specimens with a score of 0 or 1 were rated as negative. The staining intensity was equally prominent in the different samples and was therefore not integrated into the analysis. Only membranous staining was observed for RANKL.

### 2.6. Statistical Analysis

Statistical analysis was performed using SPSS26 software (IBM Germany GmbH, Ehningen, Germany). Correlations between clinical data and biomarker expression were calculated using Pearson’s Chi square test or Fisher’s exact test. A binary logistic regression analysis was used to determine the relationship between bone invasion and clinical and immunohistochemical markers. Univariate survival analysis for overall survival (OS), disease-free survival (DFS), and disease-specific survival (DSS) was calculated using the Kaplan–Meier method. Overall survival was defined as the time from diagnosis to death by any cause. Disease-free survival was determined as the time from therapy to tumor recurrence or death, whichever occurred first. Disease-specific survival was considered the time from diagnosis to tumor-related death. Median follow-up was calculated using the reverse Kaplan–Meier method. The survival distributions were compared using the log-rank test. For risk adjustment, multivariate Cox regression was applied. The results were reported with hazard ratios (HRs) and 95% confidence intervals (CIs). All reported *p*-values are two-sided and only determined as statistically significant if *p* < 0.05. As this was an exploratory study, multiple testing was not applied.

## 3. Results

The aim of this study was to determine the role of MMP-27, RANKL and OPG in bone invasion in patients with OSCC. To obtain an initial overview, we analyzed *in silico* data derived from the cBioPortal HNSCC database. Second, immunohistochemical analysis concerning the expression levels of these proteins was carried out using TMAs assembled from the bone–tumor interface of our patients’ samples. As a last step, we examined the influence of our markers, as well as the clinical and histopathological parameters, on the oncological outcome in our cohort. 

### 3.1. In Silico Data Analysis via the cBioPortal Database

**Figure 1:**
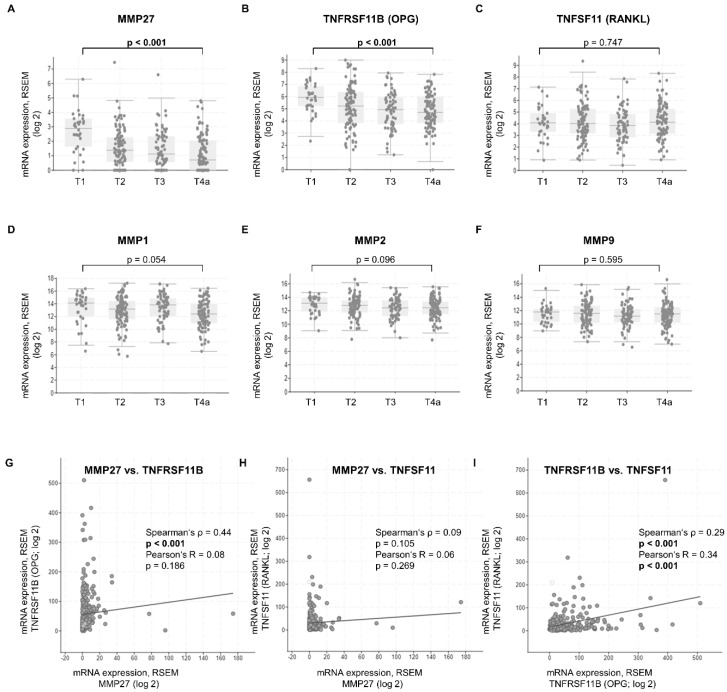
(**A**–**F**) T-stage plot of changes in mRNA expression of MMP27, TNFRSF11B (OPG), TNFSF11 (RANKL), MMP1, MMP2 and MMP9. (**A**,**B**) demonstrate a significant decline of MMP27 and TNFRSF11B expressions when comparing T1 to T4a HNSCC (*p* < 0.001). Their mRNA expression is significantly lower in T4a tumors, where invasion into nearby tissue frequently occurs. (**C**) The expression of TNFSF11 was not altered throughout the various stages. (**D**–**F**) MMP1, MMP2 and MMP9 did not show any significant associations with T-stage. N = 298, unpaired Student’s *t*-test; RSEM = relative standard error of the mean; mRNA expression normalized relative to diploid tissue. (**G**–**I**) show the correlations of mRNA expression between MMP27, TNFRSF11B and TNFSF11. (**G**) Of the 298 tumor samples provided online on cBioPortal, MMP27 mRNA expression positively correlated with TNFRSF11B (Spearman’s ρ = 0.44; *p* < 0.001; Pearson’s R = 0.08; *p* = 0.186). (**H**) No relationship was detected between MMP27 and TNFSF11 mRNA (Spearman’s ρ = 0.09; *p* = 0.105; Pearson’s R = 0.06; *p* = 0.269). (**I**) Co-expression of TNFRSF11B and TNFSF11 also demonstrated a positive correlation (Spearman’s ρ = 0.29; *p* < 0.001; Pearson’s R = 0.34; *p* < 0.001).

Results in the literature show a crucial role of MMPs, TNFRSF11B (OPG) and TNFSF11 (RANKL) concerning invasion of adjacent healthy tissue in OSCC. To obtain an initial overview of the expression of these genes in the individual T-stages, we systematically searched the cBioPortal gene database available online. We incorporated the TCGA Firehose Legacy HNSCC set into our preliminary analysis and excluded anatomical sites that did not contain OSCC. Data derived from the online database with 298 samples revealed a significant T-stage-dependent decrease of MMP27 (*p* < 0.001) and TNFRSF11B (*p* < 0.001) mRNA expression (Figure 1A,B). Comparing pT1 and pT4a, the latter demonstrating invasion into neighboring anatomical structures, showed the most significant difference. However, TNFSF11 expression did not alter significantly within the pathological Tstages (Figure 1C). With MMP1, MMP2 and MMP9, no significant differences comparing individual T-stages could be observed (Figure 1D–F). Hence, we decided to further examine MMP27 as it demonstrated a steady and highly significant decline from pT1 to pT4a, and only limited data was available in the literature. TNFRSF11B and TNFSF11 were also incorporated into our analysis because of their importance in the regulation of bone metabolism and cancer cell invasiveness. In the second step, we analyzed the gene co-expression via the cBioPortal database to further evaluate their relationship to each other. A total of 298 samples of patients with oral cancer were included. There was an evident positive correlation between MMP27 and TNFRSF11B mRNA expression (Spearman’s ρ = 0.44, Figure 1G), while TNFSF11 mRNA had no significant correlation to MMP27 (Spearman’s ρ = 0.09, Figure 1H). TNFRSF11B and TNFSF11 mRNA levels also showed a positive correlation (Spearman’s ρ = 0.29, Figure 1I).

**Figure 2:**
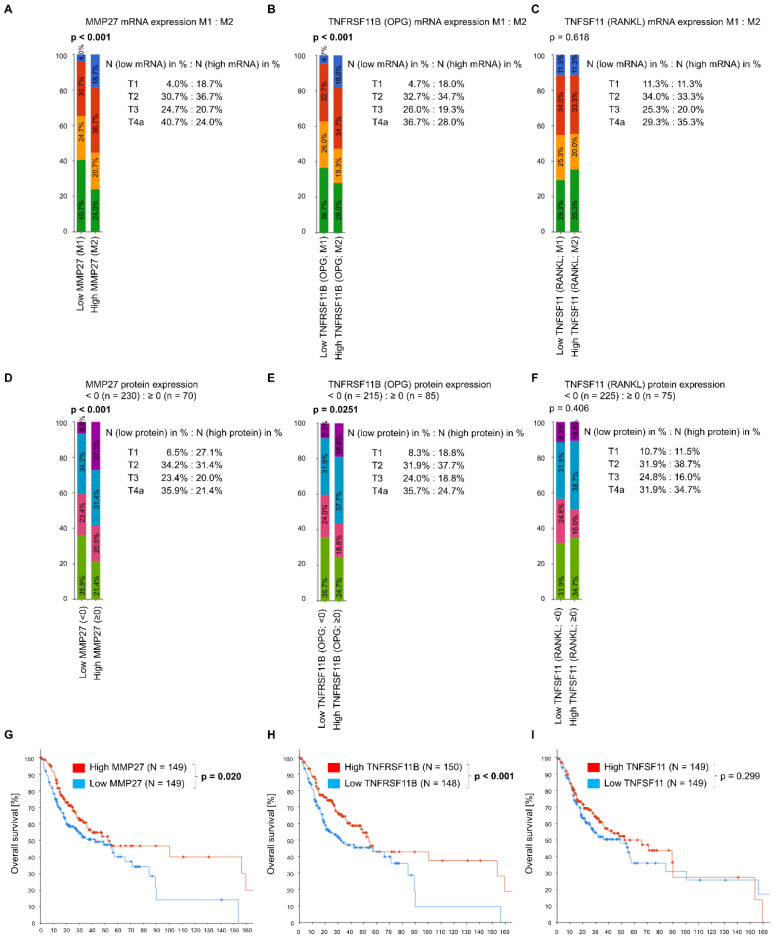
Comparison of T-stage distribution concerning the lower (M1) and the upper half (M2) of mRNA expression of MMP27, TNFRSF11B and TNFSF11 in 298 TCGA OSCC patients, based on the median. (**A**) Low MMP27 gene expression was associated with the T4a-stage compared to high MMP27 gene expression (40.7% vs. 24.0%). MRNAseq gene expression analysis from TCGA Firehose Legacy also revealed significant differential MMP27 gene expression in the AJCC T-stage code (*p* < 0.001, chi-squared test). Z-scores relative to diploid samples (log RNAseq vs. RSEM). Z-score ≤ −0.50 has been classified as low MMP27 (lower half of MMP27 mRNA expression), z-score > −0.50 was classified as high MMP27 (upper half of MMP27 mRNA expression). (**B**) Similar ratios were observed for TNFRSF11B. Here, 36.7% of patients with low expression of TNFRSF11B mRNA (M1; z-score ≤ −0.34; N = 148) presented themselves at T4a, while 28.0% of the group with high mRNA expression (M2; z-score > −0.34; N = 150) had T4a HNSCC (*p* < 0.001, chi-squared test). (**C**) No association between TNFSF11 and T-stage comparing low TNFSF11 mRNA (M1; z-score ≤ −0.32) and high TNFSF11 mRNA-expressing groups (M2; z-score > 0.32; *p* = 0.618, chi-squared test). (**D**) Looking at protein expression, samples with low MMP-27 (Z-score determined by RPPA < 0) were also associated with T4a-stage (*p* < 0.001, chi-squared test), whereas most patients with high MMP-27 expression (Z-score determined by RPPA ≥ 0) were diagnosed at an early stage. (**E**) With TNFRSF11B, the results also reflected the same ratios as for mRNA. Here, low TNFRSF11B protein expression was associated with advanced T-stage T4a (*p* = 0.0251, chi-squared test). (**F**) TNFSF11 did not show any significant association with T-stage at the protein level. (**G**,**H**) Both MMP27 and TNFRSF11B were correlated with prognosis in the cBioPortal cohort (N = 298). The two respective groups were again divided based on the median. There was a significant difference in OS when comparing low MMP27 mRNA (z-score ≤ −0.50; N = 149) to high MMP27 mRNA-expressing patients (z-score > −0.50; N = 149, *p* = 0.020, log-rank test). The same could be observed with TNFRSF11B: samples with low amounts of TNFRSF11B mRNA (z-score ≤ −0.34; N = 148) demonstrated worse OS than samples with high amounts of TNFRSF11B mRNA (z-score > 0.34; N = 150, *p* < 0.001, log-rank test). (**I**) As with T-stage analysis, no relevant effect on outcome could be demonstrated for TNFSF11. Comparison of low (z-score ≤ −0.32; N = 149) to high TNFSF11 mRNA-expressing samples (z-score > 0.32; N = 149) yielded no statistical significance (*p* = 0.299, log-rank test).

To further elucidate the correlation of MMP27, TNFRSF11B and TNFSF11 mRNA expression levels with tumor invasiveness (T4a) *in silico*, patients with low MMP27, TNFRSF11B and TNFSF11 expression (M1), as well as patients with high gene expression (M2), were selected. The two groups were divided based on the median. Thus, 298 samples could be evaluated further. Of patients with low MMP27 mRNA, 40.7% (N = 61) presented at the T4a-stage, compared to 24.0% (N = 36) with high expression of MMP27 (*p* < 0.001; Figure 2A). Moreover, more patients in the pT1-stage showed high amounts of MMP27 mRNA in their primary tumors (18.7% vs. 4.0% with low amounts of MMP27). Similar observations were made when comparing low versus high expressing TNFRSF11B samples (Figure 2B). Most patients with low TNFRSF11B mRNA levels were classified as having T4a (36.7%, N = 55). In contrast, only 41 (28.0%) of the samples expressing abundant TNFRSF11B fell into the T4a-stage. For TNFSF11, no significant associations between mRNA expression and the T4a-stage were observed (Figure 2C).

Kaplan–Meier survival analysis in the OSCC dataset revealed a significant correlation of high MMP27 (*p* = 0.020, Figure 2D) and TNFRSF11B (*p* < 0.001, Figure 2E) mRNA expression to favorable OS. The TNFSF11 mRNA content of the primary tumor was not associated with the outcome (Figure 2F).

### 3.2. Immunohistochemical Expression of MMP-27, RANKL and OPG in OSCC Tissue and Comparison to Clinico-Pathological Parameters

Inspired by the results of the *in silico* data, we decided to further analyze the expression of MMP-27, RANKL and OPG in our samples of 119 OSCCs growing in close contact with the mandibular bone (TMA cohort).

#### Patient Characteristics

Table 1 gives an overview of the clinical and histopathological characteristics of the cohort from the University Hospital of Regensburg. In summary, a total of 119 patients were included in this study; the mean age was 62.2 years (range: 38–80 years). Ninety-five patients (79.8%) were male, 24 female (20.2%). Ninety-five (79.8%) were smokers, and 88 (73.9%) consumed alcohol on a regular basis. Primary tumor sites involved the floor of the mouth (66 or 55.5%) or gingiva (53 or 44.5%); other anatomic sites were excluded from the study. Sixty-four OSCCs (53.8%) were diagnosed with pT4a-stage due to histological bone invasion, while 41 (34.5%) patients presented themselves with pT2 tumors and 14 (11.8%) with pT3 tumors, respectively. Metastatic spread to locoregional cervical lymph nodes was observed in more than half of the cohort (62 or 52.1%). G2 grade dominated among the tissue samples (96 or 80.7%), followed by G3 (18 or 15.1%) and only 5 patients presented with a G1 grade (4.2%).

### 3.3. Worst Pattern of Invasion Score and Relationship to Patient Data

**Figure 3:**
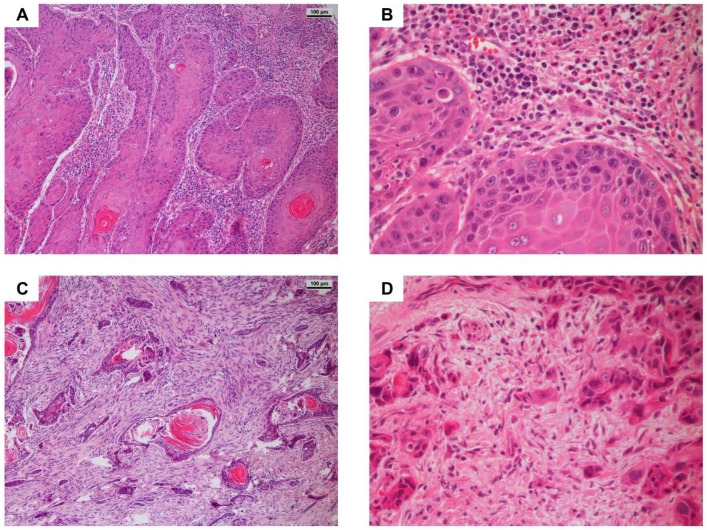
H&E-stained sections of OSCC (100× and 400× magnification). (**A**,**B**) Primary tumors classified with a score of 3, thus exhibiting a non-aggressive invasion pattern characterized by broad, finger-like pushing fronts invading surrounding tissue. (**C**,**D**) Primary tumor with a score of 4 (aggressive pattern of invasion). Here, small cell islets are detached from the main tumor, infiltrating the healthy tissue.

Many studies that have examined bone invasion in OSCC have used the WPOI grade to evaluate the pattern of invasion. To simplify the comparison of the TMA cohort with other populations in the literature, an analysis of the invasion pattern of the tumor was carried out on corresponding H&E-stained slides at the tumor invasion front (Figure 3). Sixty-one (51.3%) tumors presented a non-aggressive pattern, whereas 58 (48.7%) demonstrated aggressive behavior. Looking at the data, tumors with a WPOI grade ≥4 were more likely to show metastasis to the adjacent lymphatic tissue; however, this was not statistically significant (*p* = 0.36). In conclusion, the WPOI grade did not correlate with the clinical and histopathological data (Supplementary Table S1).

### 3.4. Immunohistochemical Staining of MMP-27, RANKL and OPG and Association with Clinico-Pathological Parameters, Particularly Bone Invasion

MMP-27, RANKL and OPG were analyzed in samples of 119 oral cancer patients (TMA cohort). The association with clinico-pathological parameters is shown in Table 1.

#### 3.4.1. MMP-27 Expression

**Figure 4:**
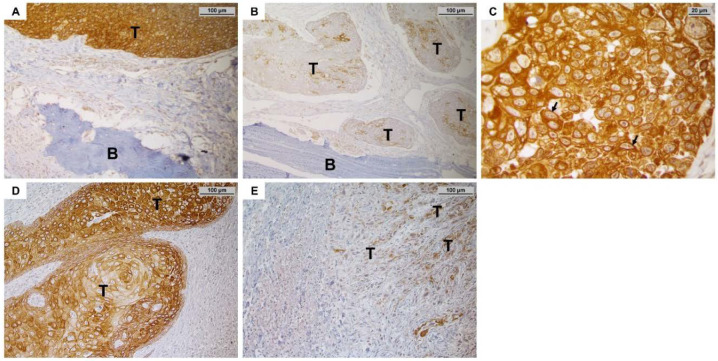
(**A**) Example of a tumor (T) with high MMP-27 expression at the bone-tumor interface (200× magnification). The tumor shows a bulky appearance and was rather distant to the adjacent bone (marked with B). (**B**) Tumor with low expression of MMP-27; the tumor mass is comprised of islets and in close proximity to the bone. (**C**) Surprisingly, the staining signal of MMP-27 was not only limited to the cytoplasm, but also detectable on the cell membrane (600× magnification). MMP-27 is usually confined to the endoplasmic reticulum. (**D**,**E**) Demonstration of the effect of MMP-27 on tumor morphology (sections without bone tissue). (**D**) This tumor shows high expression of MMP-27; the cells grow adjacent to each other. (**E**) On the other hand, this sample with low expression of MMP-27 shows single cells and clusters detached from the main tumor, which is often referred to as tumor budding.

All 119 samples revealed MMP-27 staining. Intriguingly, the signal was also located on the cell membrane (Figure 4C) and was not strictly confined to the cytoplasm. Sixty-two (52.1%) tumors showed high biomarker expression (H-score > 170.0), whereas low expression (H-score < 170.0) of MMP-27 was detectable in 57 (47.9%) samples (Table 1, Figure 4A,B), with 56.1% of pT2, 84.6% of pT3 and 43.8% of pT4a patients demonstrating high MMP27 expression. Low or absent MMP-27 staining was associated with the pT4a-stage (*p* = 0.027; 37/64 vs. 20/55), where bone invasion was present. In addition, poorly differentiated tumors classified as G3 also showed significantly lower expression of MMP-27 (*p* = 0.039; 13/18 vs. 44/101). Binary logistic regression analysis revealed low MMP-27 as the strongest predictor for the bone invasive pT4a-stage (Table 2). MMP-27 was also associated with OPG expression (Spearman’s ρ = 0.480; *p* < 0.001, Table 3). Surprisingly, while MMP-27 and RANKL expression did not correlate in our *in silico* analysis, high MMP-27 expression positively correlated with high RANKL expression in the TMA cohort (Spearman’s ρ = 0.239; *p* = 0.009).

#### 3.4.2. RANKL and OPG Expression

Further exploration of samples of tumor patients revealed that RANKL predominantly showed membranous staining (Supplementary Figure S1). Evaluation of RANKL was possible in all 119 samples. Overall, negative biomarker expression (less than 10% of cells positive) was present in 72 (60.5%) samples, while 47 (39.5%) tumors revealed positive staining for RANKL (more than 10% of cells positive). RANKL did not show any significant association between its expression and clinical or histological parameters (Table 1). 

For OPG, either cytoplasmic or nuclear staining was observed (Supplementary Figure S1). Eighty-five (71.4%) samples could be evaluated for cytoplasmic staining and 34 (28.6%) for nuclear staining (Supplementary Table S1). High cytoplasmic expression was detectable in 33 (38.8%; H-score > 40.0) specimens, whereas the majority presented with low staining positivity (52 or 61.2%; H-score < 40.0, Table 2). High cytoplasmic expression of OPG was less frequent in non-smokers compared to smokers (*p* = 0.032; 12/19 vs. 22/66). OPG expression showed a significant inverse association with younger age (≤62 years, *p* = 0.016). Additionally, high cytoplasmic staining was also associated with a more aggressive tumor phenotype, meaning a WPOI grade of ≥4 (*p* = 0.045; 20/48 vs. 14/47). The nuclear expression of OPG did not reveal any statistically significant association between expression levels and clinical or histopathological data. Binary logistic regression analysis did not provide any significant results (Table 2). However, high RANKL expression positively correlated with OPG, which is concordant to the results from cBioPortal (Spearman’s ρ = 0.381; *p* < 0.001, Table 3).

## 4. Discussion

In this retrospective study, OSCCs of the floor of the mouth and lower alveolus gingiva treated by partial or segmental mandibular resection were investigated for parameters indicating bone invasion. Tumors histologically invading mandibular bone (pT4a) were compared to tumors growing along the mandible without histological invasion (pT2, pT3). In addition to evaluation of traditional clinicopathological parameters like tumor grading and staging, histological pattern of invasion (WPOI grade 1–5) and the three different biomarkers MMP-27, RANKL and OPG were investigated for significant associations with bone invasion and patient survival. 

Currently, two different routes of infiltrative growth for mandibular bone invasion in OSCC are debated. One directly through the alveolar process and cortical bone to the cancellous bone in the edentulous mandible. In the dentate mandible, the tumor can also invade the dental socket into the cancellous part of the bone [36]. The degradation of inorganic bone compartments is complemented by the destruction and enzymatic digestion of the organic bone matrix. Matrix metalloproteinases (MMPs) are vital in degrading extracellular matrix (ECM) and are known to influence plenty of aspects in tumor cells, such as proliferation, apoptosis, invasion, angiogenesis and more [37]. Gobin et al. analyzed data from 15 different cancer types via the cancer genome atlas. Among the most elevated MMPs were MMP-1, -11, -12 and -13, while MMP-27 and -28 were mostly decreased throughout the different entities [38]. In oral cancer, multiple MMP family members have been investigated as possible biomarkers in screening or were associated with invasion or proliferation when their expression was increased [39,40,41]. Research on the oral cancer cells and tissue of OSCC patients suggests that MMP-2 and MMP-9 are frequently seen to promote infiltrative growth and bone resorption [42,43]. They are secreted by cells and require subsequent activation by proteinases [44]. In the TCGA dataset, however, MMP2 and MMP9 mRNA levels were not significantly associated with the T4a-stage. It could be possible that soluble forms of these two MMPs, which are not represented in the *in silico* data, are responsible for bone degradation.

However, the role of MMP-27 in cancer is quite unclear; to the best of our knowledge, no study has yet been done on its role in OSCC. It has been reported that a unique C-terminal domain confines MMP-27 to the endoplasmic reticulum in renal and ovarian tissue; experimental removal of this domain did not automatically lead to membrane relocation or secretion [26]. Surprisingly, we were able to detect staining signals specific to MMP-27 on the membranes of our cells, meaning that the expression was not constricted to the cytoplasm. MMP-27 could therefore be post-translationally modified in OSCC to allow it to function as a transmembranous protein. There is no further data indicating that such a modification occurs for MMP-27 in cancer, but there are reports that, for instance, MMP-14 is phosphorylated in metastatic ovarian cancer compared to its wild-type [45]. Hence, post-translational modifications are no exception and could also occur in the case of MMP-27. 

The only tumor entity in which MMP-27 has also been subject to broader research is breast cancer. Köhrmann et al. investigated tissue samples of 20 breast cancer patients. Transcriptomic analysis of MMP27 mRNA revealed low expression levels in G3 tumors compared to healthy tissue, which is consistent with our findings. However, patients demonstrating poorly differentiated tumors (G3) showed an increased presence of MMP-27, which is contrary to our results. Western blot analysis conducted by the authors revealed numerous different bands for MMP-27, which could also be non-specific or represent non-active forms of the enzyme. This complicates the interpretation [46]. Cheng et al. analyzed the GEPIA database and demonstrated that the overexpressed MMP27 gene was associated with improved OS in breast cancer. Furthermore, MMP27 expression decreased significantly in the higher stages [47]. We were also able to confirm a beneficial effect on survival in the TCGA OSCC dataset. Using RNAseq data from the cBioPortal database, we were able to demonstrate a steady T-stage-dependent decrease in MMP27 gene expression in OSCC as well. In addition, we observed a decline in MMP-27 protein expression with a higher T-stage in our patient cohort; a significant number of bone invasive pT4a-stage cancers had low MMP-27 presence compared to pT2 and pT3 OSCC. Altogether, we suggest a beneficial tumor-suppressive role for MMP-27, whereas loss of MMP-27 goes along with aggressive tumor behavior, particularly with osteolytic bone invasion. 

Another interesting result is the correlation of MMP-27 expression with RANKL or OPG expression. Analysis of *in silico* data revealed that MMP-27 and OPG mRNA were frequently co-expressed, but not MMP-27 and RANKL mRNA. At the protein level, MMP-27 and RANKL showed a positive correlation in expression in our cohort. However, it is important to keep in mind that RANKL was not associated with bone invasion, nor was it present in every tumor sample. The overall expression of RANKL in OSCC of the floor of mouth and gingiva invading mandibular bone is reported to be 75–85%, depending on the study [48,49]. Data from smaller cohorts demonstrated a positive correlation between RANKL expression, invasion pattern and poor differentiation, which is contrary to our results [50,51]. The lack of control groups or the use of different classification systems of invasion patterns impede comparison to our study. A larger cohort of 93 cancer patients revealed no significant association between RANKL and bone invasion in OSCC [52]. Moreover, several authors were able to verify RANKL expression in the stroma of the tumors, while we detected positive staining only to be present in tumor cells. RANKL expression could eventually be very dependent on the tumor microenvironment, where adjacent cells like cancer-associated fibroblasts are able to influence RANKL presence to the disadvantage of OPG expression, which could favor bone resorption [53].

On the other hand, MMP-27 and OPG demonstrated co-expression in *in silico* data, as well as in our patients. OPG has been associated with jawbone invasion in the past, where it also affected poor outcomes [52]. The presence of OPG in bone invasive OSCCs could be a sign of increased bone remodeling; a causal role of OPG for direct bone invasion has not been documented so far. Intriguingly, we were able to detect OPG expression in the nucleus of some tumor cells; the whole tumor either showed membraneous or nuclear staining. There is no documentation of translocation of OPG to the cell nucleus in the literature. We attribute this to our fixation method, which should be optimized for the OPG antibody. In addition, OPG did not influence survival in our patients, but neither did MMP-27 nor RANKL. In the online database, however, high levels of OPG mRNA prolonged the overall survival of HNSCC patients, as was the case with MMP27 mRNA. There could be a difference between the mRNA and proteomic levels. Again, post-transcriptional changes in the protein may result in the loss of effective suppression of bone invasion. Additionally, a weaker effect on prognosis in OSCC than in the whole HNSCC entity is conceivable. 

To assess the representativity of the TMA cohort compared to other OSCC cohorts in the literature, we performed a Kaplan–Meier analysis, as well as Cox regression analysis (Supplementary Figure S2, Supplementary Table S2). Another goal of the survival analysis was to determine whether the three biomarkers also influenced the prognoses of our patients. The single strongest predictor of poor OS seems to be relapse of any kind and the presence of lymph node metastasis at the time of diagnosis, which we could verify in our cohort [54]. A recently conducted meta-analysis concluded that among lymph node metastasis, perineural or lymphovascular invasion, and others, bone involvement was also an important prognostic factor [55]. Our analysis was unable to provide any other results suggesting that bone invasion influenced the outcome, but the pattern of invasion evaluated with the WPOI grade could predict poor OS. Several authors have demonstrated the prognostic impact of the WPOI grade. A WPOI grade ≥4 was highly indicative of locoregional recurrence and also correlated significantly with time to disease progression and OS in head and neck cancer patients [31,56]. We also observed low OS, with 5-year survival rates dropping from 58.3% to 43.1% when a WPOI grade of ≥4 was determined. However, WPOI failed to predict bone invasion in our cohort. This could be due to the fact that our study population excluded recurrent OSCC, which is known to be more aggressive and genetically unstable [57].

## 5. Conclusions

In summary, we suggest that MMP-27 expression functions as a tumor suppressor, whereas decreased MMP-27 expression is associated with poor differentiation and increased tumor invasiveness, particularly bone invasion, in OSCC. Increased OPG levels improve the prognosis of OSCC patients, but the exact role of OPG in mandibular bone invasion remains unclear. However, the clinical role of RANKL in mandibular invasion in OSCC remains elusive. The predominant structure of MMP-27 and its localization should be characterized in future studies.

## Figures and Tables

**Table 1:** Expression of MMP-27, RANKL and OPG and association with clinical and pathological parameters. All patients presenting with pT4a had an invasion of the mandibular bone. Significant *p*-values are highlighted in bold.

Parameter		MMP-27 (N = 119)			RANKL (N = 119)			OPG (N = 85)		
	N	Low	High	*p*-Value	Negative	Positive	*p*-Value	Low	High	*p*-Value
Age										
(Mean: 62.2 years; 38–89 years)										
>62 years	51 (42.9%)	23 (45.1%)	28 (54.9%)		33 (64.7%)	18 (35.3%)		19 (46.3%)	22 (53.7%)	
≤62 years	68 (57.1%)	34 (50.0%)	34 (50.0%)	0.711	40 (58.8%)	28 (42.2%)	0.571	32 (72.7%)	12 (27.3%)	**0.016**
Sex										
male	95 (79.8%)	44 (46.3%)	51 (53.7%)		58 (61.0%)	37 (39.0%)		42 (62.7%)	25 (37.3%)	
female	24 (20.2%)	13 (54.2%)	11 (53.0%)	0.504	15 (62.5%)	9 (37.5%)	1.000	9 (50.0%)	9 (50.0%)	0.418
Anatomic Site										
Gingiva	53 (44.5%)	26 (49.1%)	27 (50.9%)		30 (56.6%)	23 (43.4%)		24 (57.1%)	18 (42.9%)	
Floor of mouth	66 (55.5%)	31 (47.0%)	35 (53.0%)	0.855	43 (66.2%)	23 (33.8%)	0.352	27 (62.8%)	16 (37.2%)	0.661
Tobacco use										
Yes	95 (73.9%)	46 (48.4%)	49 (51.6%)		58 (61.0%)	37 (39.0%)		44 (66.7%)	22 (33.3%)	
No	24 (20.2%)	11 (45.8%)	13 (54.2%)	1.000	15 (62.5%)	9 (37.5%)	1.000	7 (36.8%)	12 (63.2%)	**0.032**
Alcohol use										
Yes	88 (73.9%)	43 (48.9%)	45 (51.1%)		54 (61.4%)	34 (38.6%)		38 (63.3%)	22 (36.7%)	
No	31 (26.1%)	14 (45.2%)	17 (54.8%)	0.835	19 (61.3%)	12 (38.7%)	0.655	13 (52.0%)	12 (48.0%)	0.467
T-stage										
pT2 + pT3	55 (46.6%)	20 (36.4%)	35 (63.6%)		36 (65.5%)	19 (34.5%)		17 (48.6%)	18 (51.4%)	
pT4a (mandibular bone invasion)	64 (53.8%)	37 (57.8%)	27 (42.2%)	**0.027**	37 (57.8%)	27 (42.2%)	0.452	34 (68.0%)	16 (32.0%)	0.115
N-stage										
N0	57 (47.9%)	27 (47.4%)	30 (52.6%)		30 (52.6%)	27 (47.4%)		23 (54.8%)	19 (45.2%)	
N+	62 (52.1%)	30 (48.4%)	32 (51.6%)	1.000	43 (69.4%)	19 (30.6%)	0.089	28 (65.1%)	15 (34.9%)	0.380
UICC-stage										
II + III	37 (28.8%)	18 (48.6%)	19 (51.4%)		21 (56.8%)	16 (43.2%)		14 (53.8%)	12 (46.2%)	
IV	82 (72.2%)	39 (47.6%)	43 (52.4%)	1.000	52 (63.4%)	30 (36.6%)	0.544	37 (62.7%)	22 (37.3%)	0.478
Grade										
G1 + G2	101 (84.9%)	44 (43.6%)	57 (56.4%)		64 (63.4%)	37 (36.6%)		41 (56.9%)	31 (43.1%)	
G3	18 (15.1%)	13 (72.2%)	5 (27.8%)	**0.039**	9 (50.0%)	9 (50.0%)	0.304	10 (76.9%)	3 (23.1%)	0.277
Tumor Recurrence										
Yes	36 (30.3%)	14 (38.9%)	22 (61.1%)		19 (52.8%)	17 (47.2%)		12 (57.1%)	9 (42.9%)	
No	83 (69.7%)	43 (51.8%)	40 (48.2%)	0.233	53 (63.9%)	30 (36.1%)	0.309	39 (60.9%)	25 (29.1%)	0.801
WPOI grade										
Non-aggressive	61 (51.3%)	29 (47.5%)	32 (52.5%)		30 (49.2%)	31 (50.8%)		33 (70.2%)	14 (29.8%)	
Aggressive	58 (48.7%)	28 (48.3%)	30 (51.7%)	1.000	33 (56.9%)	25 (43.1%)	0.464	18 (47.4%)	20 (52.6%)	**0.045**

**Table 2:** Binary logistic regression analysis of IHC markers, WPOI and histopathological grading in relation to T-stage. Significant results are highlighted in bold.

	HL		β	SE	Wald	*p*	Exp (β) (95% CI)
pT2 + pT3 vs. pT4a	0.221	High MMP27	**−0.828**	**0.392**	**4.459**	**0.035**	**0.437 (0.203–0.942)**
		RANKL	−0.182	0.415	0.192	0.662	0.834 (0.370–1.881)
		High OPG	0.349	0.427	0.670	0.413	1.418 (0.614–3.274)
		WPOI Grade 4-5	−0.146	0.402	0.132	0.717	0.864 (0.393–1.900)
		Grade	−0.307	0.566	0.295	0.587	0.735 (0.242–2.231)

HL: Hosmer-Lemeshow test (*p* < 0.05); β: regression coefficient, SE: standard error, Wald: Wald test, *p*: significance (*p* < 0.05), Exp (β) (95% CI): adjusted odds ratio (95% confidence interval).

**Table 3:** Correlation analysis of MMP-27, RANKL and OPG co-expression in our cohort. Significant results highlighted in bold.

		MMP-27	RANKL	OPG
MMP-27	Spearman’s ρ	1.000	0.239	0.480
	*p*-value	-	**0.009**	**<0.001**
	N	119	119	85
RANKL	Spearman’s ρ	0.239	1.000	0.381
	*p*-value	**0.009**	-	**<0.001**
	N	119	119	85
OPG	Spearman’s ρ	0.480	0.381	1.000
	*p*-value	**<0.001**	**<0.001**	-
	N	85	85	85

## Data Availability

The data presented in this study are available on request from the corresponding author.

## Supplementary Materials

The following supporting information can be downloaded at: https://www.mdpi.com/article/10.3390/cancers14164044/s1, Figure S1: Overview of different staining results for MMP-27, RANKL and OPG in tumor samples (200× magnification); Table S1: Table overview of patients’ characteristics and association with staining results or WPOI grade, respectively; Figure S2: Kaplan–Meier survival analysis of TMA patients; Table S2: Cox regression analysis of overall survival in TMA cohort.
