# Supplementary material for: Loss of MMP-27 Predicts Mandibular Bone Invasion in Oral Squamous Cell Carcinoma
Source: Cancers (Basel). 2022 Aug 22;14(16):4044. doi: 10.3390/cancers14164044 (PMC9406335; doi:10.3390/cancers14164044)
Supplement: Supplementary file 1 [file cancers-14-04044-s001.zip › cancers-1852835-supplementary.pdf]

Supplementary Table S1: Table overview of patients' characteristics and association with staining results or WPOI grade, respectively. Nuclear OPG and WPOI grade were not significantly associated with either clinical or histological parameters. Significant results are highlighted in bold.

**Parameter**	N = 119	MMP-27 (N = 119)			RANKL (N = 119)			OPG (N = 85)			OPG nucleus (N = 34)			WPOI grade		
		Low	High	*p*-value	Negative	Positive	*p*-value	Low	High	*p*-value	Low	High	*p*-value	Non-aggressive	Aggressive	*p*-value
Age (mean 62.2 years; 38-89 years)																
>62 years	51 (42.9%)	23 (45.1%)	28 (54.9%)		33 (64.7%)	18 (35.3%)		19 (46.3%)	22 (53.7%)		8	2		23	28	
										**0.016**	(80.0%)	(20.0%)	0.251	(45.1%)	(54.9%)	
<62 years	68 (57.1%)	34 (50.0%)	34 (50.0%)		40 (58.8%)	28 (42.2%)		32 (72.7%)	12 (27.3%)		13 (54.2%)	11 (45.8%)		38	30	
				0.711			0.571							(55.9%)	(44.1%)	0.270
Sex																
	95 (79.8%)	44 (46.3%)	51		58 (61.0%)	37 (39.0%)		42 (62.7%)	25 (37.3%)		17 (60.7%)	11 (39.3%)		49	46	
Male			(53.7%)											(51.6%)	(48.4%)	
Female	24 (20.2%)	13 (54.2%)	11 (53.0%)		15 (62.5%)	9		9	9		4	2		12	12	
				0.504		(37.5%)	1.000	(50.0%)	(50.0%)	0.418	(66.7%)	(33.3%)	1.000	(50.0%)	(50.0%)	1.000
Anatomic																
Site																
Gingiva	53 (44.5%)	26 (49.1%)	27 (50.9%)		30 (56.6%)	23 (43.4%)		24 (57.1%)	18 (42.9%)		6	5		27	26	
											(54.5%)	(45.5%)		(50.9%)	(49.1%)	
Floor of mouth	66 (55.5%)	31 (47.0%)	35 (53.0%)		43 (66.2%)	23 (33.8%)		27 (62.8%)	16 (37.2%)		15 (65.2%)	8		34	32	
				0.855			0.352			0.661		(34.8%)	0.709	(51.5%)	(48.5%)	1.000
Tobacco use																
Yes	95 (73.9%)	46 (48.4%)	49 (51.6%)		58 (61.0%)	37 (39.0%)		44 (66.7%)	22 (33.3%)		17 (58.6%)	12 (41.4%)		49	46	
														(51.6%)	(48.4%)	
No	24 (20.2%)	11 (45.8%)	13 (54.2%)		15 (62.5%)	9		7	12 (63.2%)		4	1		12	12	
				1.000		(37.5%)	1.000	(36.8%)		**0.032**	(80.0%)	(20.0%)	0.627	(50.0%)	(50.0%)	1.000
Alcohol use																
Yes	88 (73.9%)	43 (48.9%)	45 (51.1%)		54 (61.4%)	34 (38.6%)		38 (63.3%)	22 (36.7%)		18 (64.3%)	10 (35.7%)		47	41	
														(53.4%)	(46.6%)	
No	31 (26.1%)	14 (45.2%)	17 (54.8%)		19 (61.3%)	12 (38.7%)		13	12 (48.0%)		3	3		14	17	
				0.835			0.655	(52.0%)		0.467	(50.0%)	(50.0%)	0.653	(45.2%)	(54.8%)	0.532
T-Stage																
T2 + T3	55 (46.6%)	20 (36.4%)	35 (63.6%)		36 (65.5%)	19 (34.5%)		17 (48.6%)	18 (51.4%)		11	9		27	28	
											(55.0%)	(45.0%)		(50.9%)	(49.1%)	
T4a	64 (53.8%)	37 (57.8%)	27 (42.2%)		37 (57.8%)	27 (42.2%)		34 (68.0%)	16 (32.0%)		10 (71.4%)	4		33	31	
				**0.027**			0.452			0.115		(28.6%)	0.477	(51.6%)	(48.4%)	0.855
N-Stage																
N0	57 (47.9%)	27 (47.4%)	30 (52.6%)		30 (52.6%)	27 (47.4%)		23 (54.8%)	19 (45.2%)		11 (73.3%)	4		32	25	
												(26.7%)		(56.1%)	(43.9%)	
N+	62 (52.1%)	30 (48.4%)	32 (51.6%)	1.000	43 (69.4%)	19 (30.6%)	0.089	28 (65.1%)	15 (34.9%)	0.380	10 (52.6%)	9	0.296	29	33	0.360

												(47.4%)		(46.8%)	(53.2%)	
UICC Stage																
II + III	37 (28.8%)	18 (48.6%)	19 (51.4%)		21 (56.8%)	16 (43.2%)		14 (53.8%)	12 (46.2%)		8 (72.7%)	3 (27.3%)		22 (59.5%)	15 (40.5%)	
IV	82 (72.2%)	39 (47.6%)	43 (52.4%)	1.000	52 (63.4%)	30 (36.6%)	0.544	37 (62.7%)	22 (37.3%)	0.478	13 (56.5%)	10 (43.5%)	0.465	39 (47.6%)	43 (52.4%)	0.242
Grade																
G1 + G2	101 (84.9%)	44 (43.6%)	57 (56.4%)		64 (63.4%)	37 (36.6%)		41 (56.9%)	31 (43.1%)		19 (65.5%)	10 (34.5%)		53 (52.5%)	48 (47.5%)	
G3	18 (15.1%)	13 (72.2%)	5 (27.8%)	**0.039**	9 (50.0%)	9 (50.0%)	0.304	10 (76.9%)	3 (23.1%)	0.277	2 (60.0%)	3 (40.0%)	0.348	8 (44.4%)	10 (55.6%)	0.613
Tumor Recurrence																
Yes	36 (30.3%)	14 (38.9%)	22 (61.1%)		19 (52.8%)	17 (47.2%)		12 (57.1%)	9 (42.9%)		12 (80.0%)	3 (20.0%)		18 (50.0%)	18 (50.0%)	
No	83 (69.7%)	43 (51.8%)	40 (48.2%)	0.233	53 (63.9%)	30 (36.1%)	0.309	39 (60.9%)	25 (29.1%)	0.801	9 (47.4%)	10 (52.6%)	0.079	43 (51.8%)	40 (48.2%)	1.000
WPOI grade																
Non-aggressive	61 (51.3%)	29 (47.5%)	32 (52.5%)		30 (49.2%)	31 (50.8%)		33 (70.2%)	14 (29.8%)		10 (71.4%)	4 (28.6%)				
Aggressive	58 (48.7%)	28 (48.3%)	30 (51.7%)	1.000	33 (56.9%)	25 (43.1%)	0.464	18 (47.4%)	20 (52.6%)	**0.045**	11 (55.0%)	9 (45.0%)	0.477			

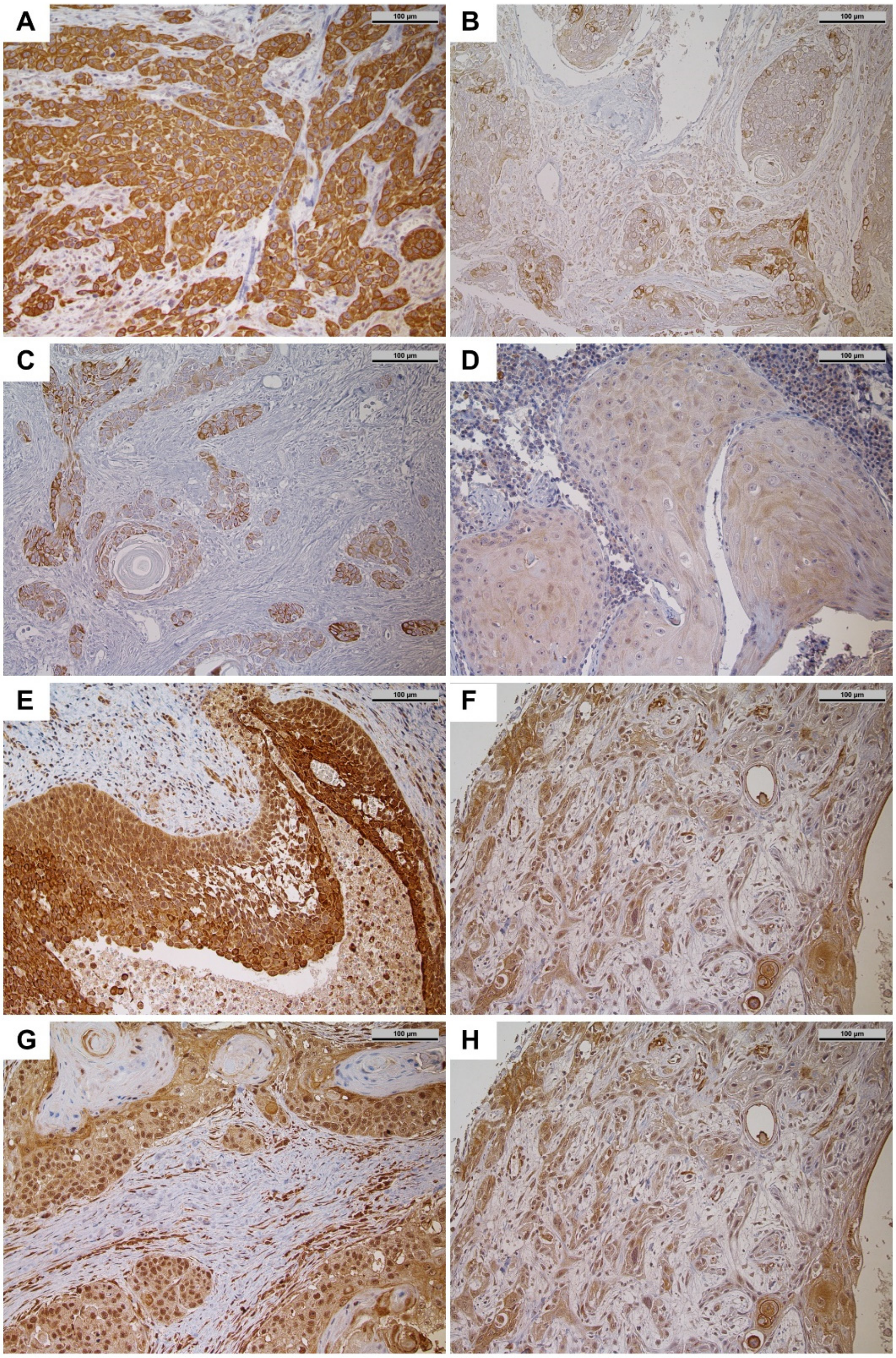

Supplementary Figure S1: Overview of different staining results for MMP-27, RANKL and OPG in tumor samples (200x magnification). **A** and **B** MMP-27 exclusively exhibited membranous staining. **A** High expression of MMP-27, determined as H-Score of 300. **B** Low expression MMP-27, rated with a maximum H-Score of 40. **C** and **D** RANKL only showed membranous staining. **C** OSCC with positive RANKL expression, determined as 3 or more than 50%. **D** OSCC with negative RANKL expression, determined as 1 or less than 10%. **E**–**H** OPG demonstrated either cytoplasmic (**E**,**F**) or nuclear (**G**,**H**) staining. **E** High expression of cytoplasmic OPG, rated with an H-Score of 285. **F** Low cytoplasmic expression of OPG, determined as H-Score of 50. **G** For comparison, a tumor with high nuclear expression of OPG, determined as H-Score of 180. **H** OSCC with low nuclear expression of OPG, rated with an H-Score of 35.

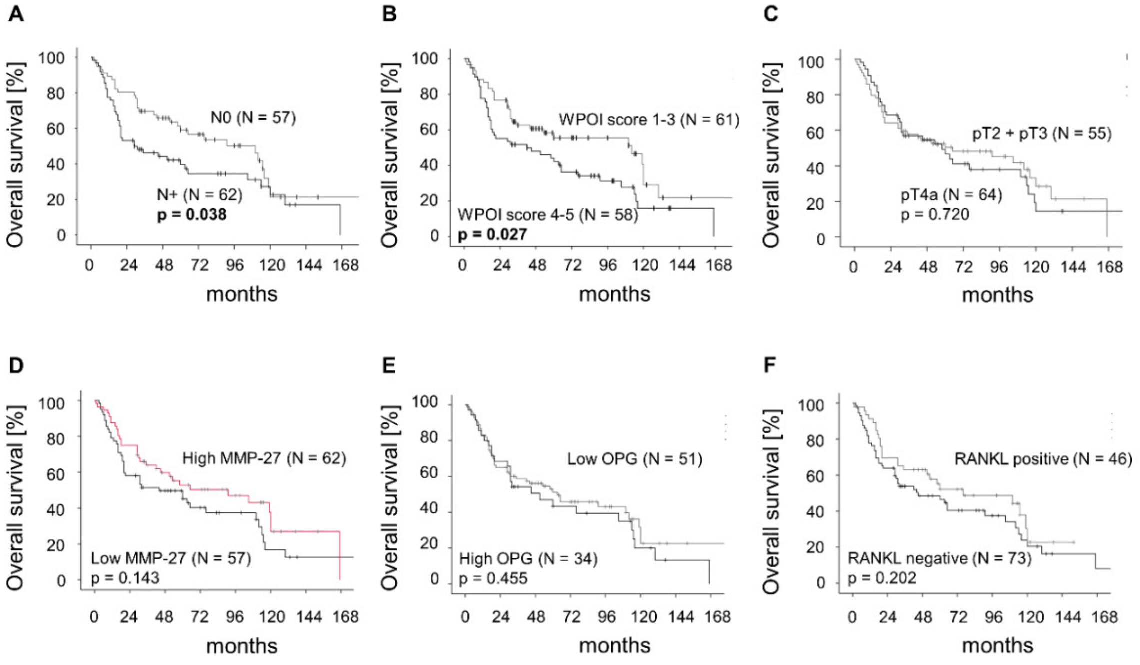

Supplementary Figure S2: Overview of Kaplan-Meier survival analysis of the TMA cohort. **A** and **B** As described in the text, metastasis to cervical lymph node predicted worse OS in the TMA cohort (p = 0.038) **B** A high WPOI grade of ≥ 4 was also indicative of unfavorable OS (p = 0.027). **C** Comparing pT4a to pT2 and pT3 together, no effect on outcome could be demonstrated. Patients presenting with pT4a had a slightly worse OS, although not statistically significant (p = 0.720) **D–F** Neither MMP-27 nor OPG or RANKL had an impact on outcome in the TMA cohort.

Supplementary Table S2: OS analysis of TMA patients using univariate and multivariate Cox regression analysis. Relapse of any kind was a significant predictor for OS in uni- and multivariate analysis. HR = Hazard ratio; CI = Confidence interval. Significant results are highlighted in bold.

	**Univariate Cox regression**			**Multivariate Cox regression**		
	p	**HR**	**95% CI**	***p***	**HR**	**95% CI**
High MMP27	0.148	0.712	0.449–1.129			
RANKL	−0.182	0.207	0.454–1.187			
High OPG	0.460	1.198	0.742–1.935			
T4a	0.689	0.907	0.563–1.461			
WPOI grade 4-5	**0.030**	**1.673**	**1.052–2.659**			
N+	**0.042**	**1.619**	**1.018–2.576**			
Relapse (any)	**<0.001**	**20.018**	**4.906–81.686**	**<0.001**	**19.305**	**4.698–79.325**
